# Sonographic Predictors and Outcomes of Second-Dose Misoprostol in Early Pregnancy Loss

**DOI:** 10.7759/cureus.93241

**Published:** 2025-09-25

**Authors:** Nadine Ashkar Majadla, Lital Shiloach, Inshirah Sgayer, Ala Aiob, Lior Lowenstein, Marwan Odeh

**Affiliations:** 1 Raya Strauss Wing Department of Obstetrics and Gynecology, Galilee Medical Center, Nahariya, ISR; 2 Azrieli Faculty of Medicine, Bar Ilan University, Safed, ISR

**Keywords:** anembryonic pregnancy, delayed miscarriage., early pregnancy loss, misoprostol efficacy, missed abortion, surgical intervention

## Abstract

Background: Misoprostol is a preferred medical agent for managing early pregnancy loss. However, the optimal protocol, particularly repeat dosing, remains a topic of debate.

Objective: To evaluate the effectiveness of a second dose of misoprostol in women with incomplete uterine evacuation after the first dose, and to examine sonographic findings and demographic data to identify factors associated with successful treatment.

Methods: Retrospective descriptive study of electronic medical records from a tertiary university-affiliated hospital. We identified women who had early pregnancy loss and received a second dose of misoprostol vaginally after an incomplete response to the first dose.

Results: A second dose of misoprostol achieved a 49% success rate for uterine evacuation without surgical intervention. Demographic and obstetric characteristics were similar in both the intervention and non-intervention groups; 44% of delayed miscarriages and 49% of anembryonic pregnancies with an intact sac persisted after the second dose (p<0.001). Delayed miscarriage and anembryonic pregnancy after the first dose significantly increased the odds of surgical intervention (delayed miscarriage: odds ratio (OR) 4.138, 95% confidence interval (CI) 1.513-11.317; anembryonic pregnancy: OR 2.921, 95% CI 1.349-6.326).

Conclusion: A second dose of misoprostol offers effective care. Sonographic results after the first dose help forecast the need for surgical intervention.

## Introduction

Early pregnancy loss is defined as the loss of an intrauterine pregnancy before the completion of the 12th week of gestation, confirmed by ultrasound evidence of either the absence of fetal heart activity or a lack of increase in crown-rump length (CRL) over one week, or the persistent presence of an empty gestational sac [1]. Delayed miscarriage, also known as missed abortion, is defined as the unrecognized intrauterine death of the embryo or fetus without being naturally expelled, constituting about 15% of clinically diagnosed pregnancies [2].

Approaches for early pregnancy loss include expectant management, medical treatment, and surgical intervention. Surgical therapy for delayed miscarriage has been performed worldwide for 50 years [3]. However, the costs and risks of complications remain concerns. Intrauterine adhesions caused by surgery may reduce fertility, making expectant or medical management more suitable options [4,5]. However, expectant management has unpredictable success rates of 25-76% [6-8] and can cause wasted time and anxiety [6], leading to increased risks of emergency surgical treatment and blood transfusion [9].

Misoprostol (CYTOTEC®), a prostaglandin E1 analog, is commonly used in obstetrics and gynecology for its safety and efficacy in expelling nonviable pregnancy tissue, thereby avoiding surgery and its attendant risks. The optimal route and dosage of misoprostol for delayed miscarriage remain unclear. The National Institute for Health and Care Excellence recommends a single 800-microgram dose of misoprostol, administered vaginally or orally [10].

The recommended initial dose of misoprostol by the American College of Obstetricians and Gynecologists (ACOG) in the treatment of delayed miscarriage is 800 mcg, administered vaginally. The ACOG permits one repeat dose of misoprostol in cases of incomplete uterine evacuation following the first dose [11]. However, no consensus exists in the literature regarding the appropriate criteria to define the complete expulsion of pregnancy tissue without a gestational sac, and various endometrial thickness values have been utilized across studies. Furthermore, the role of repeat misoprostol administration among women with a thickened endometrium is not well established. Our study aimed to evaluate the efficacy of a second dose of misoprostol in women with incomplete uterine evacuation due to early pregnancy loss following the first dose and to predict factors that may affect treatment success.

## Materials and methods

Study description and population 

We conducted a retrospective cohort study at a university-affiliated hospital from January 2016 to November 2023, reviewing electronic medical records of women with early pregnancy loss who received 800 mcg of vaginal misoprostol. Eligible women required a second dose of misoprostol due to retained products of conception, identified by follow-up transvaginal sonography. Criteria for incomplete evacuation included a thickened endometrium over 20 mm, persistent gestational material, or other signs of incomplete evacuation. Clinical symptoms such as continued bleeding prompted sonographic evaluation. Women receiving a second dose due to retained products post-delivery or after an incomplete/septic abortion were excluded, as were those needing urgent surgical evacuation for severe complications. The study was approved by the Institutional Review Board of Galilee Medical Center (Approval number 0195-21-NHR).

Ultrasound studies 

A gynecologist conducted an ultrasound panel for early first-trimester miscarriage diagnosis and ruled out a viable intrauterine pregnancy. Transvaginal sonography revealed the type of early pregnancy loss, diagnosing a delayed miscarriage per the American College of Obstetricians and Gynecologists (ACOG) guidelines. Now more commonly termed an anembryonic pregnancy, a condition known as a blighted ovum, was characterized by minimal embryonic debris without heart rate activity [12]. A thickened endometrium, greater than 20 mm, suggested retained products of conception. Additional sonographic findings included gestational material in the cervix and an irregular endometrium. Vaginal misoprostol was administered, followed by routine transvaginal ultrasounds to monitor uterine evacuation within one week. These evaluations aimed to detect retained products and determine if a second misoprostol dose was needed, which was usually assessed during the second ultrasound on the same day.

Treatment of the early pregnancy loss protocol

This study includes only women who chose the pharmacological option for managing early pregnancy loss and adapted to this management according to the departmental protocol. Second doses of misoprostol 800 mcg were administered vaginally. Four tablets of 200 mcg each were placed in the posterior fornix of the vagina by the attending physician. Women were asked to lie down for 5 to 10 minutes and were hemodynamically monitored for at least 30 minutes following misoprostol administration. Side effects were reported.

Study outcomes

Treatment success was defined as complete expulsion of gestational tissue after the second dose of misoprostol, confirmed by both clinical assessment and transvaginal ultrasound showing an empty uterine cavity or endometrial thickness of ≤20 mm without retained products. Treatment failure was defined as the continued presence of gestational tissue on ultrasound after the second misoprostol dose, which required surgical evacuation. The primary outcome of the study was the efficacy of a second misoprostol dose in early pregnancy loss, determined by the need for surgical intervention. Secondary outcomes included the timing of misoprostol doses, the interval between doses, sonographic findings after each dose, and the need for analgesics, intravenous or oral iron supplementation, and hospitalization. Data on subsequent pregnancies and the time to next pregnancy were collected when available.

Calculations

Statistical Analysis

We reported continuous variables as means ± standard deviations or medians and ranges. Qualitative variables are presented as frequencies and percentages. Continuous variables were compared using the independent sample t-test or the Mann-Whitney test based on the groups' sample sizes and the distribution shapes of the variables. Categorical variables were analyzed using Pearson's chi-squared test or Fisher's exact test. A multivariable logistic regression model was performed to examine correlations of demographic, clinical, and sonographic variables with the likelihood of need for intervention. A two-tailed p-value <0.05 was considered statistically significant. The data were analyzed with SPSS Statistics software, version 27 (IBM Corp., Armonk, NY).

Sample Size Calculation

Previous studies established an initial treatment success rate of 70-80% [13,14]. Based on existing literature and clinical observations, we hypothesized a 20% effectiveness for a second dose of misoprostol. Our calculated sample size was 120 women at a significance level of 0.05, with a study power of 99%. Although we collected data from 152 women, 141 were included in the study. The sample size was determined using IBM SPSS Statistics software, version 27.

## Results

**Figure 1:**
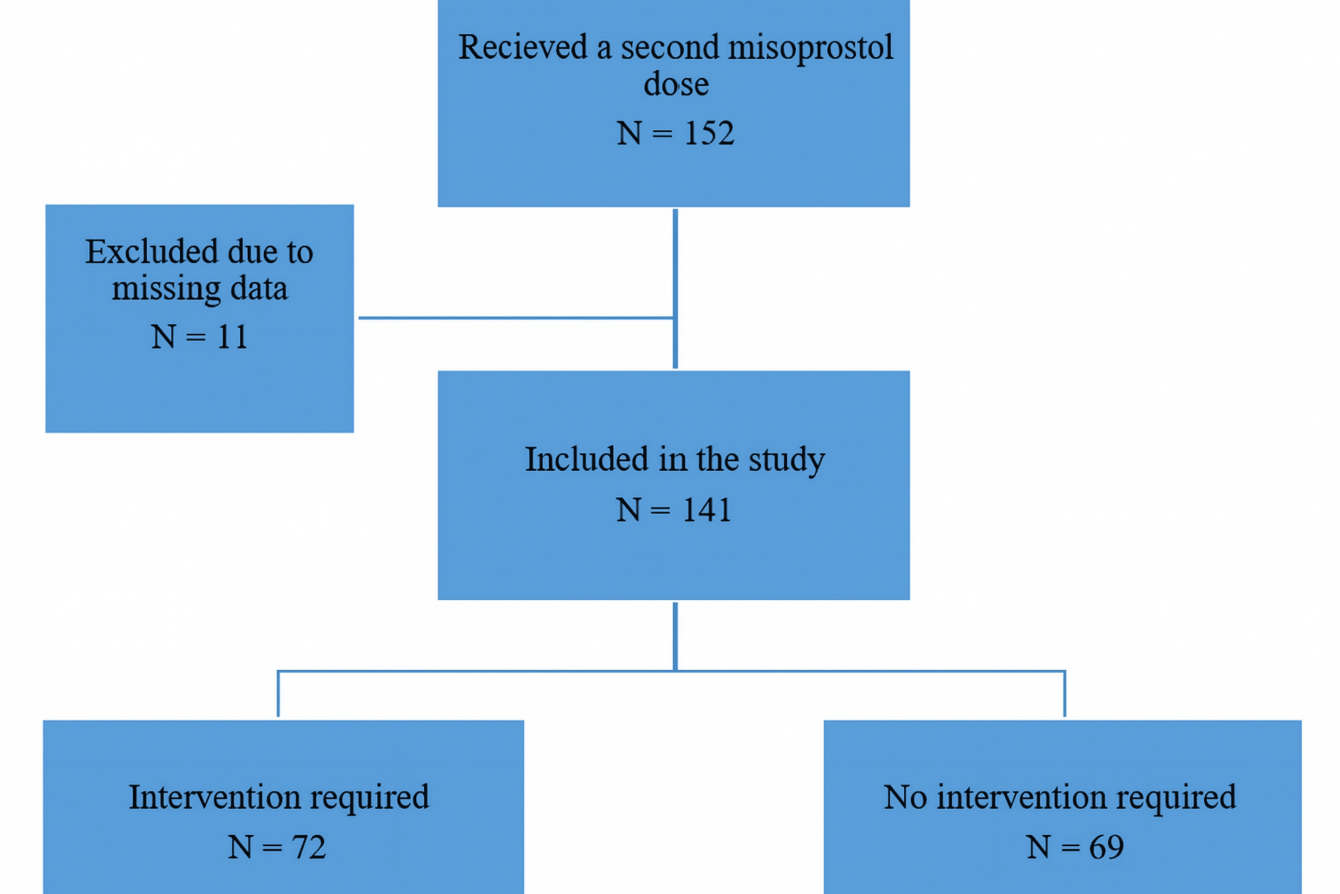
Flow chart of the study population

During the study, 1060 women received the first misoprostol dose; 152 (14%) received a second. Of these, 49% (69 women) succeeded without surgical intervention (Figure 1). Most pregnancies were spontaneous, with four achieved via in vitro fertilisation (IVF). Surgical interventions were primarily based on sonographic findings, although one woman required intervention for heavy bleeding due to retained products of conception, confirmed by a thickened endometrium. She was stable and needed intravenous iron supplementation. Demographic characteristics were similar between groups, with a higher percentage of Jewish women requiring interventions (55% vs. 45%; p = 0.04). There were no emergencies requiring urgent surgical evacuation, and three women needed iron for low hemoglobin, while one required iron and a blood transfusion. No significant differences were noted in obstetric history or misoprostol use, and no serious complications occurred following the second misoprostol dose (Table 1).

**Table 1:** Characteristics of the patients The data are presented as number (%), mean + standard deviation or median (X) *Chi-Square Test, **Fisher's Test, +T-Test, ++ Mann-Whitney Test

		No intervention required n=69	Intervention required n=72	P value
Ethnicity	Arab	42 (62)	32 (45)	*0.042 Df=1 Effect size =0.177
	Jew	25 (37)	39 (55)	
Age (years)		29.5±6.9 30 (17-44)	29.69±5.8 29 (19-45)	+0.895
Weight (kg)		67.1±15 65 (41-124)	67.7±13.3 67.5 (45-107)	+0.832
Height (cm)		162.9±6.3 162 (150-180)	164.6±6.5 164.5 (148-180)	+0.149
BMI (kg/m2)		25.2±4.9 24.2 (16-43)	24.9±4.5 24.5 (18-42)	+0.726
Smoking		2 (3)	2 (3)	**1.000
Chronic disease		10 (14)	13 (18)	*0.651 Df=1 Effect size =0.048
Chronic medications		4 (5)	7 (9)	*0.533 Df=1 Effect size =0.073
Gravidity		2 (1-7)	2 (1-9)	++0.759
Number of previous vaginal births		0 (0-4)	0 (0-4)	++0.848
Number of previous cesarean sections		0 (0-2)	0 (0-2)	++0.581
Previous early pregnancy loss		0 (0-2)	0 (0-4)	++0.434
Previous Spontaneous abortion		8 (40)	10 (62)	**0.373
Previous Delayed miscarriages (missed abortion)		8 (40)	3 (18)	**0.373
Previous Termination of pregnancy		4 (20)	3 (18)	**0.373
Gestational age (weeks)		9.7±1.8	9.4±1.4	+0.353
Hemoglobin level before first dose (mg/dl)		12.7 (9.3-14.5)	12.5 (8.2-14.3)	+0.249
The interval between first and second doses (days)		2 (1-18)	2 (1-12)	++0.815
Time of administering analgesics after the second dose (days)		0 (0-6)	0 (0-4)	++0.288
Administration of oral or intravenous iron		0	3 (4)	**0.245
Duration of hospitalization (days)		3 (0-5)	3 (0-6)	++0.282
Next pregnancy				
Failed		5 (12)	5 (13)	++1.000
Ongoing		1 (2)	(00)	++1.000
Successful delivery		36 (86)	34 (87)	++1.000

Use of analgesia after misoprostol

The frequency of analgesic administration among patients is categorized into two groups: intervention and no-intervention. Following the first dose of misoprostol, the median analgesic usage was higher in the intervention group; however, the difference observed between the two groups was not statistically significant (p=0.578). After the second dose, most patients in both groups did not require any analgesics; similarly, the difference in analgesic use remained non-significant (p=0.201) (Table 2).

**Table 2:** Analgesic use after misoprostol ++ Mann-Whitney Test

		No intervention required n=69													Intervention required n=72													p-value
Analgesics after the first dose		0 (0-5)													1 (0-5)													0.578
	Times (n)	0		1		2		3		4		5		6	0		1		2		3		4		5		6	
	Patients (n)	35		13		14		3		1		3		0	35		22		9		4		0		2		0	
Analgesics after the second dose		0 (0-6)													0 (0-4)													0.201
	Times (n)	0	1		2		3		4		5		6		0	1		2		3		4		5		6		
	Patients (n)	46	12		9		1		0		0		1		54	10		5		2		1		0		0		

Sonographic findings and the need for intervention after the first dose

Following the first dose of misoprostol, the sonographic findings differed between women who did and did not require intervention. A majority (n=37, 53%) of those who did not need intervention were diagnosed with a thickened endometrium, 22 (32%) were identified as having an anembryonic pregnancy, and eight (12%) showed sonographic evidence of a delayed miscarriage. Conversely, 33 (45%) of those who needed intervention were diagnosed with an anembryonic pregnancy and 19 (26%) with a thickened endometrium; 24% exhibited sonographic signs of a delayed miscarriage (p<0.001) (Table 3).

**Table 3:** Clinical and sonographic findings after the first and second doses of misoprostol The data are presented as numbers (%) **Fisher's Test

Sonographic findings	After the first dose		After the second dose	
	No intervention required n=69	Intervention required n=72	No intervention required n=69	Intervention required n=72
Delayed miscarriage	8 (12)	17 (24)	0	7 (10)
Anembryonic pregnancy	22 (32)	33 (46)	0	27 (37)
Thickened endometrium	37 (54)	19 (26)	1 (1)	22 (31)
Products of conception in the cervix	1 (1)	0	0	0
Thickened endometrium and positive Doppler exam	1 (1)	2 (3)	0	8 (11)
Irregular apparent endometrium	0	1 (1)	68 (99)	8 (11)
	**P<0.001		**P<0.001	

Sonographic findings and intervention needed after the second misoprostol dose

Following the second dose of misoprostol, the sonographic findings distinguished between women who required intervention and those who did not. The majority, 68 (98%), of those who did not require intervention displayed an irregular apparent endometrium sonographically, while one (1%) exhibited a thickened endometrium. Delayed miscarriages and having an anembryonic pregnancy did not occur after the second dose of misoprostol, which was deemed successful. In contrast, among those who required intervention, 27 (37%) had an anembryonic pregnancy, 22 (30%) presented a thickened endometrium, eight (11%) exhibited a thickened endometrium with positive color Doppler flow, and another eight (11%) demonstrated an irregular apparent endometrium. Seven (10%) in this group showed sonographic evidence of a delayed miscarriage with a complete intrauterine gestational sac and embryo (p<0.001). Of the eight women, two had borderline endometrial thickness-19 mm and 19.5 mm, resulting in surgical intervention. The other six exhibited thicknesses under 20 mm after the second dose, but follow-up revealed retained products of conception, necessitating hysteroscopy (Table 3).

Types of early pregnancy loss requiring intervention

**Figure 2:**
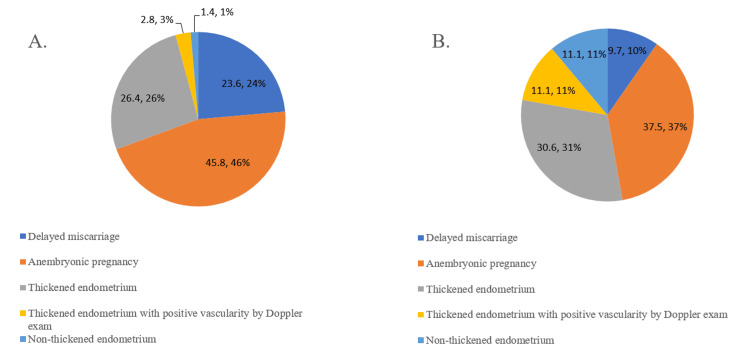
Distributions of reasons for early pregnancy loss after the first and second dosages of misoprostol among women requiring intervention A. After the first dose of misoprostol; B. After the second dose of misoprostol.

Among the women who needed intervention, those who received one dose had higher rates of delayed miscarriage (24% vs. 10%) and anembryonic pregnancy (46% vs. 37%), as well as a lower rate of thickened endometrium (26% vs. 31%) compared to those who received two doses. Most women who did not require intervention showed a thickened endometrium (53%) after the first dose. In contrast, after the second dose, irregular endometrium was observed in most cases (98%) (see Figure 2).

Factors affecting the need for intervention

The number of previous vaginal births and gravidity did not correlate with the need for intervention: odds ratio (OR) 0.918, 95% CI 0.507-1.660, p=0.776, and OR 1.018, 95% CI 0.650-1.593, p=0.939, respectively. Likewise, the interval between the first and second doses did not correlate with the need for intervention: OR 0.918, 95% CI 0.787-1.070, p = 0.272. However, the sonographic findings, delayed miscarriage, and an anembryonic pregnancy were associated with the need for intervention: OR 4.138, 95% CI 1.513-11.317, p<0.001, and OR 2.921, 95% CI 1.349-6.326, p<0.001, respectively (Table 4).

**Table 4:** Correlations between obstetric and treatment parameters and the need for intervention

		Significance	Odds Ratio	95% Confidence Interval	
				Lower	Upper
Gravidity		0.94	1.02	0.65	1.59
Number of previous vaginal births		0.78	0.92	0.50	1.66
The interval between the first and second doses (days)		0.27	0.92	0.79	1.07
Sonographic findings after the first dose		0.005			
	Delayed miscarriage	0.006	4.14	1.5	11.3
	Anembryonic pregnancy	0.007	2.92	1.35	6.3

## Discussion

This study contributes to the ongoing debate about the efficacy and effectiveness of a second misoprostol dose for managing early pregnancy loss. The topic of administering a repeat dose of misoprostol is addressed in guidelines from several international societies. The Royal College of Obstetricians and Gynaecologists (RCOG) recommends considering an additional dose of misoprostol if women do not experience bleeding within 24 hours of administration or do not follow the prescribed medication regimen [15]. The WHO guideline states the timing between misoprostol doses in days rather than hours and does not specify a maximum number of doses [16]. The ACOG suggests using 800 µg of vaginal misoprostol for early pregnancy loss, with the option of administering a repeat dose no earlier than three hours after the first and within seven days if there is no response. ACOG reports that the overall success rate was approximately 84% when a second 800 µg vaginal dose is administered if needed, while in our cohort, the success rate of uterine evacuation after a second dose of misoprostol is 49% [11].

We found that the number of previous vaginal births and the total number of pregnancies did not show a significant correlation with the need for intervention. This finding differs from the previous study by Odeh et al., which indicated that a history of one or more previous deliveries was associated with an increased failure of misoprostol treatment in early pregnancy loss [17]. Our analysis identified ethnicity as the only significant demographic factor influencing the success of the second misoprostol dose. Specifically, a higher proportion of Jewish women required surgical intervention compared to their Arab counterparts. These findings are supported by Muhsen et al., who provided information regarding the inequality of health systems among the major populations in Israel (Arabs and Jews), including access to follow-up care. Arabs in Israel have exhibited lower healthcare utilization rates and worse health outcomes across various clinical areas [18]. Recognizing the disparities between Arabs and Jews must be considered. Efforts to improve equitable access to ultrasound and standardized counseling may help reduce variations in care and ensure that women are offered repeat medical management when appropriate.

This study reveals that 49% of women who received a second dose of misoprostol did not require intervention. In comparison, Mizrachi et al. reported a success rate, defined as no intervention required, of 77% for a second misoprostol dose, compared to 76% for those who received a single dose (RR 0.98; 95% CI 0.83-1.16; p=0.89) [13]. The difference in successful outcomes can be attributed to the trial approach, where participants were selected similarly and the population was chosen homogeneously. Our study, in contrast, is characterized by grouping most types of early pregnancy loss with sonographic findings that are clinically relevant and commonly encountered. The diagnostic finding of endometrial thickness (>20 mm) in our study, which was not necessarily a successful finding in Mizrachi et al.'s study (< 15 mm), is a crucial aspect of our research [13]. Zhang et al. demonstrated that a second dose yielded a 60% success rate. However, they defined treatment as successful only if there was complete expulsion using a 30 mm cut-off, sparking debates about the best criteria for determining treatment success [14].

Managing thickened endometrium (>20 mm) remains challenging. Among 56 women with thickened endometrium after the first dose, 27% retained this condition after the second dose, and none exhibited a regular endometrial appearance. Our study showed that a thickened endometrium after one dose was common in women who did not require intervention; however, neither thickened endometrium after the first dose nor after the second dose can predict the need for intervention. In a study by Rottenstreich, a repeat dose of misoprostol administered to women with residual endometrial thickness after initial medical treatment for early pregnancy failure does not lead to lower rates of subsequent surgical interventions. Furthermore, no correlation was found between increased endometrial thickness and treatment outcomes in cases of thickened endometrium [19].

The optimal timing for follow-up and the administration of the second misoprostol dose remains uncertain. Current guidelines suggest a repeat dose within seven days if there is no response [11]. However, we found no significant differences regarding the interval between the first and second doses and the success of misoprostol treatment. While the median time between doses was consistent at two days in both groups, the range varied between women who required intervention and those who did not: 1-12 and 1-18 days, respectively. Our study design differs from that of both Zhang et al. [14] and Mizrachi et al. [13], who predetermined the interval between doses as two and four days, respectively. As previously mentioned, our success rate after the second dose was lower than that reported by Zhang et al. However, their results may not be universally applicable, and some clinicians propose that the efficacy of misoprostol treatment is also influenced by the time interval to follow-up. Higher success rates have been reported when clinicians wait longer before assessing success or failure [14]. Exploring a larger cohort may yield more significant results.

We have limited findings regarding the future fertility of women who received a second dose of misoprostol during early pregnancy loss. Our study showed no significant difference in the likelihood of a successful subsequent pregnancy between women who needed intervention and those who did not (47% vs. 52%, p=1). Bord et al. indicated that misoprostol treatment for first-trimester delayed miscarriage does not negatively impact future fertility, particularly for those with a positive reproductive history who are younger than 35 years old [20].

An important aspect of our study is the association of the sonographic findings after the first dose of misoprostol with the success rate of the second dose. Following the initial administration of misoprostol, women whose sonographic findings indicated delayed miscarriage and an anembryonic pregnancy were at a higher risk of requiring intervention, by 4, 4.1, and 2.9 times, respectively, compared to those who exhibited a thickened endometrium. Our findings suggest that after the initial misoprostol dose, the presence of sonographic findings such as delayed miscarriage and an anembryonic pregnancy significantly increases the likelihood of necessitating surgical intervention. The clinical significance of this observation is that these sonographic findings can serve as valuable parameters in patients' decision-making processes when choosing among various methods for treating delayed miscarriage.

Our study has limitations, including its retrospective nature and the data collected over the years based on keywords, potentially introducing selection bias. Additionally, the single-center setting may limit the generalizability of the findings. Inter-observer variability represents a common limitation in sonographic studies. The management of thickened endometrium following miscarriages remains a topic of debate, as it is uncertain whether conservative or interventional approaches are more appropriate, given that not all cases indicate residual tissue. The threshold for thickened endometrium that necessitates surgical intervention or conservative management needs further investigation. Moreover, utilizing thickened endometrium as an indicator for retained products of conception might present a risk of overtreatment, as this finding does not consistently indicate the presence of retained products. Consequently, this may lead to unnecessary surgical interventions for patients who might otherwise experience spontaneous resolution through expectant management. These circumstances highlight the need for further research within this specific patient population. One limitation is that the study provided data solely for a misoprostol-only treatment group, without comparing it to other commonly used treatment options for managing early pregnancy loss. We have limited data on follow-up and subsequent pregnancies as well as fertility outcomes, which could have provided valuable insights into the long-term effects of treatment. Future studies and investigations are required.

## Conclusions

Our study revealed a 49% success rate for achieving uterine cavity evacuation in women who received a repeated misoprostol regimen. Sonographic findings, particularly delayed miscarriages and anembryonic pregnancies, significantly predicted the need for surgical intervention. Increased treatment success is observed among those presenting with a thickened endometrium. These results hold essential significance in clinical discussions with women considering medical treatment options for early pregnancy loss, taking into account factors that may potentially increase the likelihood of surgical intervention. Additionally, clinicians may consider sonographic findings after the first dose when counseling patients about the potential need for surgical intervention. Although our trial found that sonographic findings can indeed help diagnose residuals after miscarriages, it is important to emphasize that additional predictors are needed to prevent untreated residuals and unnecessary interventions. Further investigations are required, and future guidelines could benefit from including considerations, such as recognizing disparities in access to ultrasound, follow-up, and patient counseling, to promote more consistent and patient-centered care.
